# Office Blood Pressure and Arteriolosclerosis in Biopsy‐Proven Glomerulonephritis: Association With Renal Function Decline and Clinical Outcomes

**DOI:** 10.1111/jch.70367

**Published:** 2026-09-19

**Authors:** Antonietta Gigante, Alice Trombacco, Giulia Galanti, Chiara Pellicano, Georgia Sorato, Rosa Cascone, Edoardo Rosato, Konstantinos Giannakakis, Rosario Cianci

**Affiliations:** ^1^ Department of Translational and Precision Medicine Sapienza University of Rome Rome Italy; ^2^ Department of Radiological, Oncological and Anatomo‐Pathological Sciences, Pathological Anatomy Sapienza University of Rome Rome Italy

**Keywords:** blood pressure, kidney disease, proteinuria, renal biopsy, renal function

## Abstract

Hypertension is a major determinant of chronic kidney disease progression, yet the relationship between standardized office blood pressure measurements (OBPM), chronic vascular lesions, and prognosis in biopsy‐proven glomerulonephritis (GN) remains poorly defined. We investigated the association between OBPM and renal histopathological lesions, focusing on arteriolosclerosis, and assessed their prognostic significance. This retrospective longitudinal study included 66 adults with biopsy‐proven GN. All patients underwent standardized OBPM, laboratory evaluation, and renal biopsy. Histopathological assessment included glomerular, vascular, tubular, and interstitial lesions. Patients were followed for 36 months. Mixed‐effects models evaluated longitudinal changes in blood pressure, proteinuria, and estimated glomerular filtration rate (eGFR), while logistic regression identified predictors of renal and cardiovascular outcomes. Patients with arteriolosclerosis had significantly higher systolic blood pressure (SBP) than those without (130 vs. 120 mmHg, *p* = 0.032). SBP > 130 mmHg was associated with arteriolosclerosis (*p* = 0.004), arterial intimal fibrosis (*p* = 0.005), crescentic lesions (*p* = 0.033), and glomerulosclerotic changes (*p* = 0.048), whereas no associations were observed for diastolic blood pressure (DBP). During follow‐up, proteinuria significantly decreased (*p* < 0.001), whereas SBP remained stable. Arteriolosclerosis was associated with a steeper decline in eGFR over time (interaction *β* = −4.91 mL/min/1.73 m^2^ per follow‐up interval, *p* = 0.023) and independently predicted the composite renal and cardiovascular outcome (OR 6.67, 95% CI 1.01–44.31; *p* = 0.049). In biopsy‐proven GN, higher SBP is closely associated with chronic intrarenal vascular lesions, particularly arteriolosclerosis. Arteriolosclerosis independently predicts renal function decline and adverse cardiorenal outcomes, supporting its value as a histopathological marker for risk stratification and individualized blood pressure management.

## 1 Introduction

Glomerulonephritis (GN) encompasses a heterogeneous group of immune‐mediated disorders characterized by inflammation and injury of the glomeruli and represents one of the leading causes of chronic kidney disease (CKD) and end‐stage kidney disease (ESKD) worldwide. Clinical presentation ranges from isolated urinary abnormalities to nephrotic syndrome, nephritic syndrome, acute kidney injury (AKI), and progressive CKD. Despite substantial advances in the understanding of disease pathogenesis and the development of targeted therapeutic strategies, the clinical course of GN remains highly heterogeneous, reflecting the complex interplay between immune‐mediated injury, chronic structural damage, and modifiable cardiovascular risk factors [1, 2].

Renal biopsy remains the gold standard for the diagnosis and classification of GN, providing essential histopathological information for disease characterization, prognostic stratification, and therapeutic decision‐making [2, 3, 4]. Besides defining the underlying glomerular lesion, renal biopsy allows the assessment of chronic vascular and tubulo‐interstitial changes, including arteriolosclerosis, arterial intimal fibrosis, tubular atrophy, and interstitial fibrosis, which represent irreversible structural damage and are increasingly recognized as important determinants of long‐term renal outcome [4, 5, 6]. These chronic lesions reflect the cumulative effects of sustained hemodynamic and inflammatory injury and may influence disease progression independently of the underlying glomerular disease.

Hypertension is one of the most important modifiable determinants of CKD progression, promoting vascular remodeling, nephron loss, and progressive renal dysfunction, while impaired kidney function further exacerbates hypertension, creating a vicious cycle of cardiorenal damage. Reflecting the central role of blood pressure control in CKD, current guidelines recommend intensive treatment, although systolic blood pressure (SBP) targets differ slightly between the KDIGO, ESH, and ESC recommendations [7, 8, 9].

Despite the recognized role of hypertension in CKD progression, the relationship between elevated office blood pressure measurement (OBPM) and chronic vascular lesions in biopsy‐proven GN remains poorly characterized. In particular, it is still unclear whether higher OBPM is associated with arteriolosclerosis and whether this lesion provides independent prognostic information for renal function decline and adverse renal and cardiovascular outcomes.

Therefore, the aim of the present study was to investigate the association between OBPM and chronic histopathological lesions in patients with biopsy‐proven GN, with particular emphasis on arteriolosclerosis. Secondary objectives were to evaluate the longitudinal changes in blood pressure, proteinuria, and kidney function during follow‐up, and to assess the prognostic value of chronic vascular lesions for renal function decline as well as renal and cardiovascular outcomes.

## 2 Methods

This retrospective longitudinal observational study included consecutive adult patients with biopsy‐proven GN who underwent native kidney biopsy at Policlinico Umberto I, “Sapienza” University of Rome, and were subsequently followed on an outpatient basis at the same institution. Exclusion criteria were known renal artery stenosis, urinary tract obstruction, heart failure, incomplete baseline clinical, laboratory, or histopathological data, and unavailable follow‐up information. Overall, 66 patients fulfilled the study criteria and were included in the analysis.

Clinical and laboratory data were collected at the time of renal biopsy and at each scheduled follow‐up visit. The assessment included clinical evaluation and routine laboratory investigations, including standardized OBPM [7], serum creatinine (sCr), urinalysis, and 24 h proteinuria. Estimated glomerular filtration rate (eGFR) was calculated using the Chronic Kidney Disease Epidemiology Collaboration (CKD‐EPI) equation [10].

Patients were followed for 36 months after renal biopsy. Clinical and laboratory evaluations were performed at baseline and after 12, 24, and 36 months. At each visit, OBPM, sCr, eGFR, 24 h proteinuria and clinical outcomes were recorded.

Histopathological evaluation was performed according to previously published and validated criteria adopted by our group [11]. Briefly, glomerular, vascular, tubular, and interstitial lesions were systematically assessed by two experienced renal pathologists blinded to clinical and laboratory data. The evaluated lesions included segmental and global glomerulosclerosis, fibrinoid necrosis, crescents, arteriolosclerosis, arteriolar hyalinosis, arterial intimal fibrosis, tubular atrophy, interstitial fibrosis, and interstitial inflammation.

### 2.1 Study Outcomes

The primary objective of the study was to investigate the association between OBPM and histological renal lesions. Secondary objectives included the evaluation of the longitudinal evolution of BP, proteinuria, and renal function, as well as the prognostic significance of chronic vascular lesions.

Renal outcome was defined as the occurrence of kidney replacement therapy (dialysis or kidney transplantation) or a significant decline in renal function during follow‐up. A composite outcome including both renal and cardiovascular events was also evaluated. Cardiovascular events were defined as acute myocardial infarction or stroke occurring during follow‐up.

The study was conducted according to the principles of the Declaration of Helsinki and was approved by the local Ethics Committee. Written informed consent for renal biopsy and the use of anonymized clinical data for research purposes was obtained from all participants.

### 2.2 Statistical Analysis

After evaluation of normality distribution with Shapiro‐Wilk test, continuous variables were expressed as mean ± standard deviation (SD) or median and interquartile range (IQR). Categorical variables were reported as absolute numbers and percentages. Comparisons between groups were performed using the Mann–Whitney U test for continuous variables and chi‐square or Fisher's exact test for categorical variables, as appropriate. Baseline associations between OBPM and histological lesions were assessed considering both SBP and diastolic blood pressure (DBP) as continuous variables, as well as categorical variables using clinically relevant thresholds (SBP >120 mmHg, SBP >130 mmHg and SBP > 140 mmHg). Correlations between BP values and clinical/laboratory variables were evaluated using Spearman's rank correlation analysis.

Longitudinal changes in SBP, DBP, proteinuria, and eGFR during follow‐up (baseline, 12, 24, and 36 months) were analyzed using mixed‐effects models for repeated measures. Interaction analyses were performed to assess whether baseline histological lesions influenced the longitudinal behavior of blood pressure and proteinuria over time.

Renal outcome was defined as the occurrence of dialysis initiation, kidney transplantation, or significant renal function deterioration. Exploratory analyses were also performed using a composite clinical outcome including both renal and cardiovascular events. Logistic regression analyses were conducted to identify variables associated with outcomes. Due to the limited number of events, multivariable models were kept parsimonious and considered exploratory.

A two‐sided *p* value <0.05 was considered statistically significant. Statistical analyses were performed using SPSS 25.

## 3 Results

### 3.1 Study Population

Sixty‐six patients with biopsy‐proven GN were included in the study. The cohort comprised 39 (59.1%) females with a mean age of 52.7 ± 17.3 years. Baseline mean eGFR was 59.1 ± 31.7 mL/min/1.73 m^2^, while median 24 h proteinuria was 2.6 g/day (IQR 0.8–6.4). Mean SBP and DBP were 132.2 ± 21.8 mmHg and 79.9 ± 11.1 mmHg, respectively. Histological examination revealed a relevant prevalence of chronic vascular and tubulo‐interstitial lesions. Arteriolosclerosis (45.6%), fibrointimal arterial fibrosis (40.4%), tubular atrophy (80.4%), and interstitial fibrosis (68.4%) were frequently observed.

Baseline demographic, clinical, laboratory, and histological characteristics of the study population are summarized in Table 1.

**TABLE 1:** Baseline characteristics of the study population.

Variable	Mean ± SD	Median (IQR)	*n* (%)
Age, years	52.7 ± 17.5	54.00 (39.25–66.00)	
Sex: F			39 (59.1)
SBP, mmHg	132.2 ± 21.8	130.00 (120.00–140.00)	
DBP, mmHg	79.9 ± 11.1	80.00 (70.00–85.00)	
eGFR, ml/min/1.73m^2^	59.1 ± 31.7	55.00 (35.00–84.00)	
Creatinine, mg/dL	1.7 ± 1.3	1.30 (0.90–1.70)	
Proteinuria, g/24h	4.3 ± 4.7	2.60 (0.80–6.41)	
Albumin, g/dL	3.0 ± 1.0	3.20 (2.29–3.88)	
Renal outcome			16 (24.2)
Composite outcome			6 (9.1)
SBP >140 mmHg			12 (18.2)
SBP >130 mmHg			40 (61.5)
SBP >120 mmHg			53 (81.5)
Primary glomerular diseases			47 (71.2)
‐ Membranous nephropathy			19 (28.8)
‐IgA nephropathy			12 (18.2)
‐FSGS			11 (16.7)
‐Other primary GN			5 (7.6)
Secondary/systemic glomerular diseases			19 (28.8)
– ANCA‐associated GN			6 (9.1)
– Lupus nephritis			3 (4.5)
‐Crioglobulinemic GN			3 (4.5)
– Other secondary glomerular diseases			7 (10.6)
Histology: Segmental sclerosis			17 (32.1)
Histology: Global glomerulosclerosis			14 (26.4)
Histology: Any glomerulosclerosis			12 (22.6)
Histology: Fibrinoid necrosis			5 (9.6)
Histology: Crescents			14 (26.4)
Histology: Arteriolosclerosis			26 (45.6)
Histology: Arterial intimal fibrosis			23 (40.4)
Histology: Arteriolar hyalinosis			25 (43.9)
Histology: Tubular atrophy			45 (80.4)
Histology: Casts			31 (55.4)
Histology: Hyaline droplets			30 (53.6)
Histology: Interstitial edema			1 (1.8)
Histology: Interstitial fibrosis			39 (68.4)
Histology: Interstitial inflammation			38 (69.1)

**Abbreviations**: ANCA, anti‐neutrophil cytoplasmic antibodies; BP, blood pressure; DBP, diastolic Blood Pressure; eGFR, estimated Glomerular Filtration Rate; FSGS, focal segmental glomerular sclerosis; GN, glomerulonephritis; SBP, Systolic Blood Pressure.

### 3.2 Baseline Associations Between Office Blood Pressure and Histological Lesions

**FIGURE 1:**
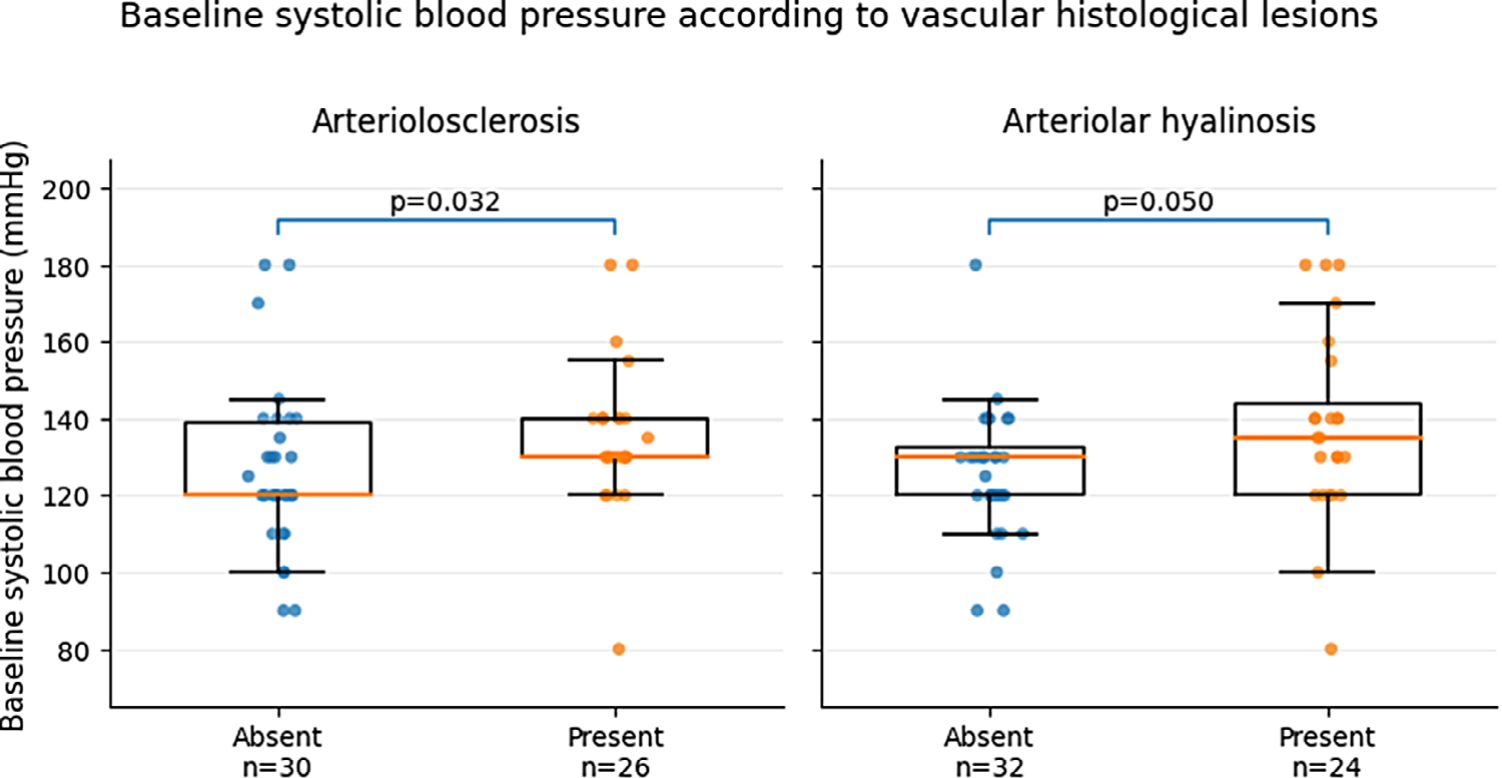
Baseline SBP according to histological lesions.

Patients with arteriolosclerosis showed significantly higher SBP values compared with those without arteriolosclerosis (median 130 mmHg [IQR 130–140] vs 120 mmHg [IQR 120–138.8], *p* = 0.032). Similarly, patients with arteriolar hyalinosis tended to have higher SBP values than those without hyalinosis (135 mmHg [IQR 120–143.8] vs 130 mmHg [IQR 120–132.5], *p* = 0.050). These results are shown in Figure 1.

**FIGURE 2:**
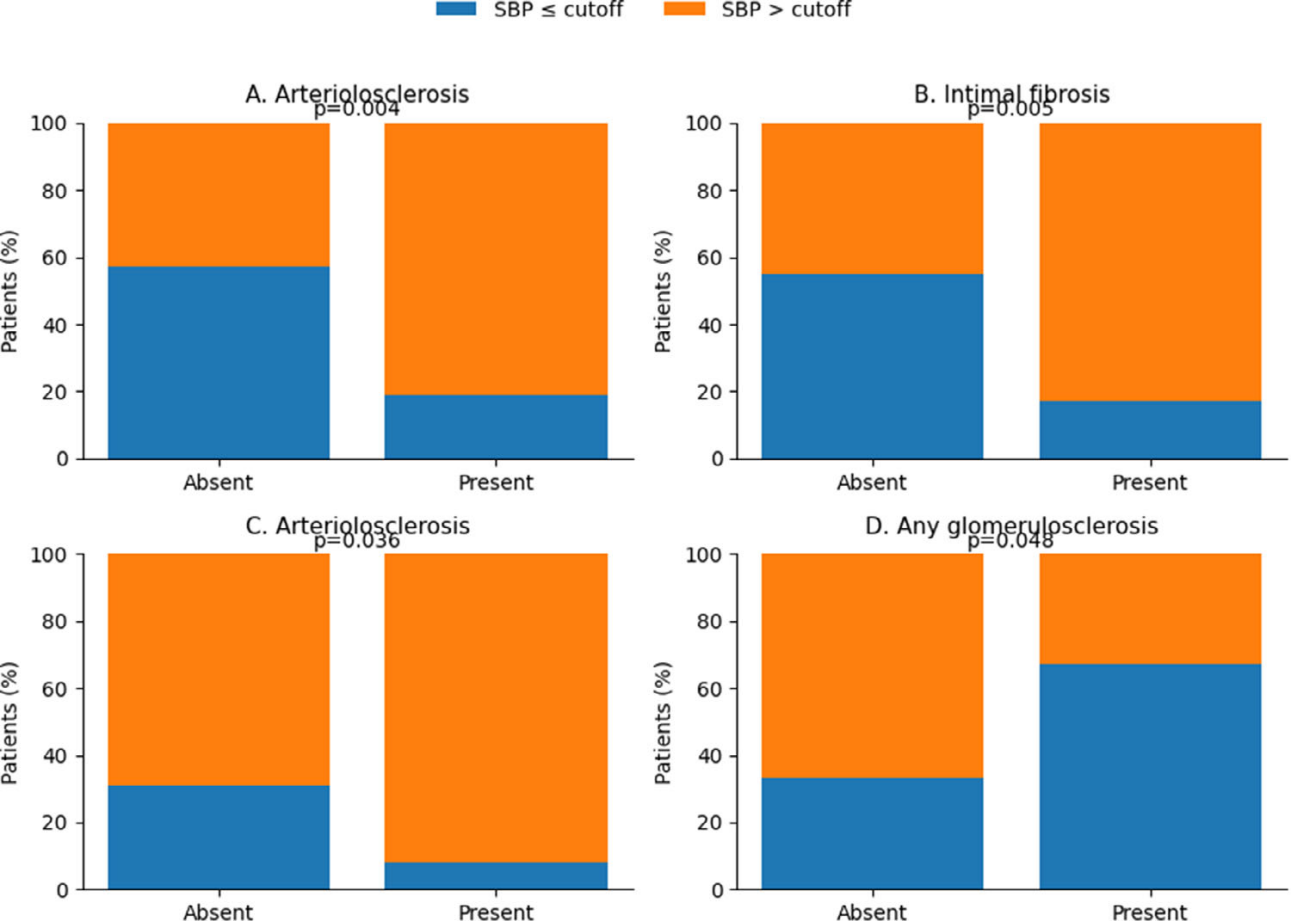
Association between SBP tresholds and histological lesions.

An SBP >130 mmHg was significantly associated with arteriolosclerosis (21/26 patients with arteriolosclerosis vs 13/30 without arteriolosclerosis; *χ* ^2^ *p* = 0.004) and arterial intimal fibrosis (19/23 vs 15/33; *χ* ^2^ *p* = 0.005). In addition, SBP >130 mmHg was associated with crescentic lesions (5/14 vs 26/38; *χ* ^2^ *p* = 0.033). Using a lower threshold, SBP >120 mmHg remained significantly associated with arteriolosclerosis (24/26 vs 21/30; *χ* ^2^ *p* = 0.036). Finally, SBP >130 mmHg was significantly associated with any histological evidence of glomerulosclerotic changes (Fisher's exact test, *p* = 0.048). These results are shown in Figure 2. No significant associations were observed between DBP and most histological parameters.

Only 12 (18.2%) patients had SBP > 140 mmHg. Using this more selective SBP threshold, arteriolosclerosis was present in 4/12 (33.3%) of patients with SBP > 140 mmHg, compared with 22/54 (40.7%) of those with SBP ≤ 140 mmHg (*p* = 0.751). The composite renal and cardiovascular outcome occurred in 41.7% versus 31.5%, respectively (*p* = 0.515); these findings should be interpreted cautiously because of the limited number of patients with SBP >140 mmHg.

### 3.3 Longitudinal Analysis

**FIGURE 3:**
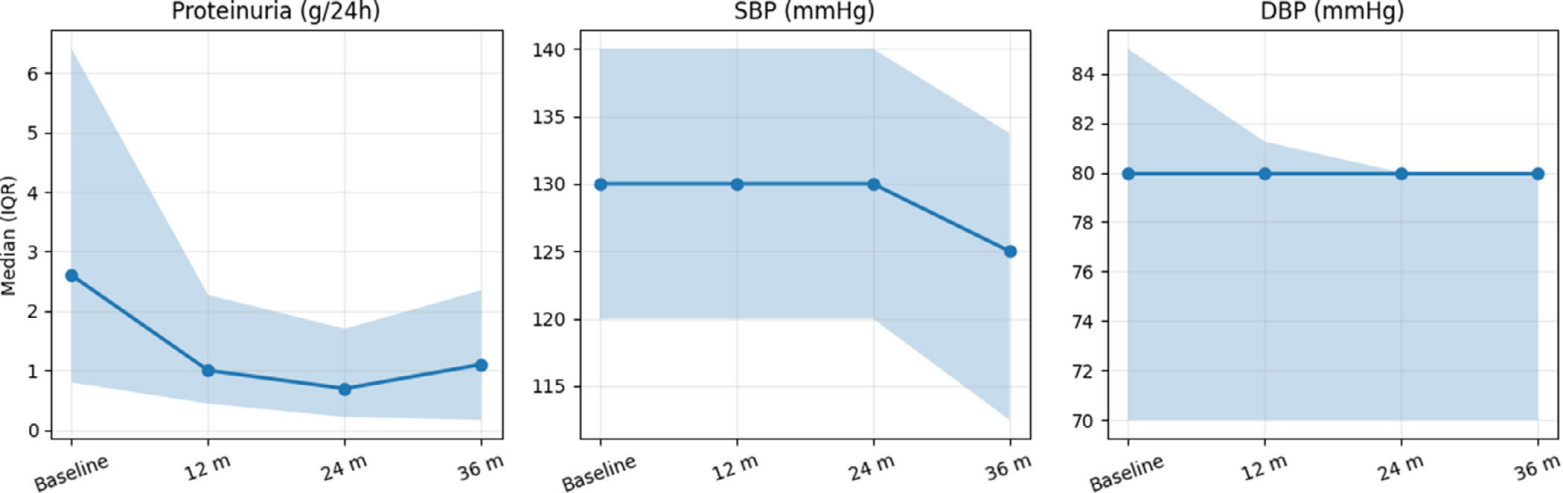
Longitudinal variation of proteinuria, SBP and DBP overt time.

During follow‐up, proteinuria significantly decreased over time (*β* = −0.229, *p* < 0.001) of 0.23 g/24 h per visit and DBP showed a mild but significant reduction during follow‐up (*β* = −1.564, *p* = 0.017) with a reduction of 1.6 mmHg for each visit, whereas SBP remained relatively stable throughout the observation period (*β* = −1.205, *p* = 0.241) with a tendency towards a reduction of 1.2 mmHg for each visit (Figure 3). However, neither baseline arteriolosclerosis nor the interaction between arteriolosclerosis and time significantly influenced proteinuria evolution (group effect *p* = 0.583; interaction *p* = 0.774).

**FIGURE 4:**
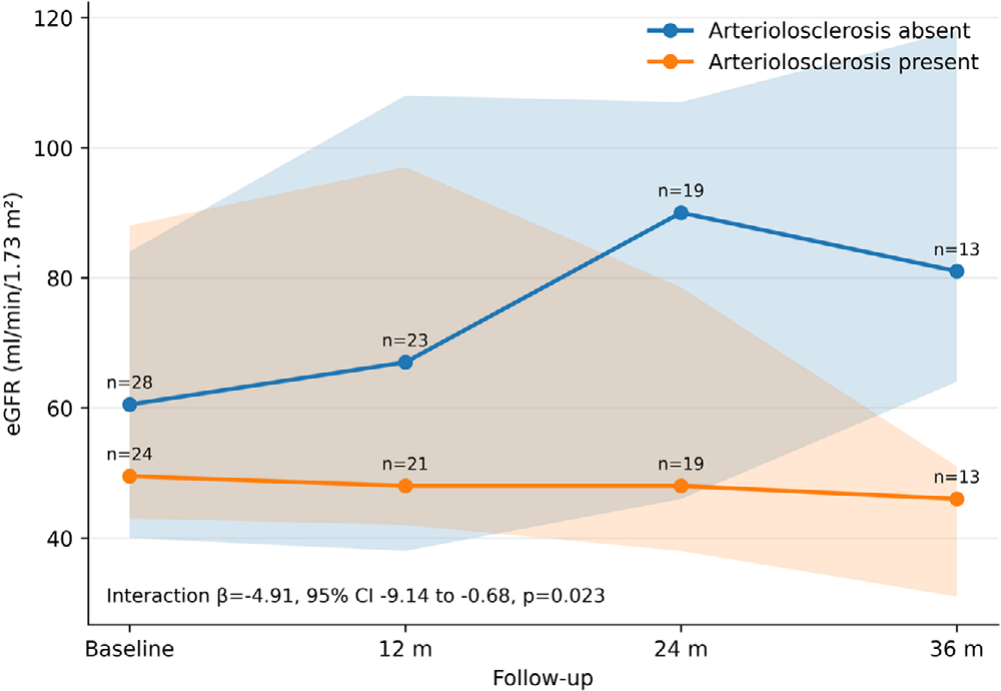
Longitudinal variation of eGFR according to presence of arteriolosclerosis.

Patients with arteriolosclerosis showed a less favorable longitudinal eGFR trajectory compared with those without arteriolosclerosis. At baseline, median eGFR was 49.5 mL/min/1.73 m^2^ (IQR 43.0–88.0) in patients with arteriolosclerosis and 60.5 mL/min/1.73 m^2^ (IQR 40.0–84.0) in those without. During follow‐up, eGFR remained substantially stable or declined in the arteriolosclerosis group, reaching 46.0 mL/min/1.73 m^2^ (IQR 31.0–51.0) at 36 months, whereas patients without arteriolosclerosis showed higher eGFR values at 36 months (81.0 mL/min/1.73 m^2^, IQR 64.0–118.0). In the mixed‐effects model, the interaction between arteriolosclerosis and time was significant (*β* = −4.91 mL/min/1.73 m^2^ per follow‐up step, 95% CI −9.14 to −0.68; *p* = 0.023) (Figure 4).

### 3.4 Outcome Analysis

During follow‐up, renal outcome events occurred in 16 patients, whereas 6 additional patients experienced cardiovascular events. In multivariable logistic regression analysis adjusted for age, baseline eGFR, proteinuria, and clinicopathological diagnosis (primary versus secondary/systemic glomerular disease), arteriolosclerosis remained independently associated with the composite renal and cardiovascular outcome (OR 6.92, 95% CI 1.02–47.12; *p* = 0.048) (Table 2). Clinicopathological diagnosis (OR 1.35, 95% CI 0.22–8.42; *p* = 0.746), age (OR 0.99, 95% CI 0.92–1.06; *p* = 0.708), eGFR (OR 1.01, 95% CI 0.98–1.04; *p* = 0.437) and proteinuria (OR 1.99, 95% CI 0.73–5.42; *p* = 0.180) were not independently associated with the composite outcome (Table 2).

**TABLE 2:** Multivariable logistic regression analysis for composite outcome.

Variables	OR (95% CI)	*p*
Primary vs secondary/systemic GN	1.35 (0.22−8.42)	0.746
Age	0.99 (0.92–1.06)	0.708
Arteriolosclerosis	6.92 (1.02–47.12)	0.048
eGFR	1.01 (0.98–1.04)	0.437
Proteinuria	1.99 (0.73–5.42)	0.180

Abbreviations: CI, confidence interval; GN, glomerulonephritis; OR, odds ratios; eGFR, estimated glomerular filtration rate.

## 4 Discussion

The present study demonstrates a significant association between higher office SBP and chronic vascular lesions, particularly arteriolosclerosis, in patients with biopsy‐proven GN. Furthermore, the presence of arteriolosclerosis at the time of kidney biopsy was associated with a less favorable longitudinal trajectory of renal function and independently predicted adverse renal and cardiovascular outcomes, supporting its role as a marker of disease severity and long‐term prognosis.

Our findings further suggest that SBP is more closely related to chronic intrarenal vascular remodeling than DBP. Notably, the association between SBP and arteriolosclerosis remained significant even when a threshold of 120 mmHg was applied and became even stronger above 130 mmHg. These observations support the concept that chronic vascular damage develops along a continuum of SBP values rather than only in the presence of overt hypertension, highlighting the potential clinical relevance of even modest elevations in SBP in patients with GN.

Chronic exposure to elevated SBP promotes structural remodeling of the intrarenal arterioles, characterized by vascular wall thickening and luminal narrowing. These changes impair renal autoregulation, increase intraglomerular pressure, reduce tissue perfusion, and ultimately contribute to chronic glomerular ischemia and progressive kidney injury [12].

Consistent with these mechanisms, Hill et al. [13] demonstrated that remodeling of the renal afferent arterioles leads to focal impairment of autoregulation, resulting in heterogeneous glomerular hemodynamics within the same kidney. This heterogeneous vascular remodeling exposes some glomeruli to increased systemic pressure while others undergo chronic ischemia, providing a structural explanation for the coexistence of glomerulosclerosis and chronic ischemic lesions observed in hypertensive kidney disease.

The resulting imbalance between glomerular hypertension and chronic ischemia represents a major mechanism driving nephron loss and CKD progression [12].

Our findings are consistent with previous observations by Kohagura et al. and Hill et al. [12, 13] supporting the concept that chronic vascular remodeling impairs renal autoregulation and contributes to progressive kidney dysfunction. The close association between higher SBP and arteriolosclerosis observed in our cohort suggests that elevated SBP may not simply reflect the presence of renal damage but may actively contribute to chronic vascular remodeling within the kidney. In turn, these vascular lesions may amplify susceptibility to further blood pressure‐dependent renal injury by disrupting autoregulatory mechanisms and promoting progressive glomerular and tubulointerstitial damage.

This concept is further supported by growing evidence indicating that biomarkers linked to endothelial dysfunction and microvascular injury, particularly serum uric acid, are associated with glomerular ischemic lesions and chronic vascular damage in patients with glomerular diseases. These observations further strengthen the hypothesis that endothelial injury represents a key mechanism linking elevated SBP to chronic vascular remodeling and progressive renal dysfunction [11, 12, 13, 14].

A particularly relevant finding of our study is that patients with arteriolosclerosis experienced a significantly less favorable longitudinal trajectory of renal function. Although proteinuria significantly decreased during follow‐up and SBP remained relatively stable, the presence of arteriolosclerosis was associated with a progressive decline in eGFR over time, suggesting that chronic vascular lesions identify a component of renal injury that is not fully captured by conventional clinical markers.

Chronic exposure to elevated SBP promotes structural remodeling of the intrarenal vasculature, characterized by arteriolar hyalinosis, medial hypertrophy, and luminal narrowing. These vascular changes impair the autoregulatory capacity of the afferent arteriole, thereby increasing susceptibility to glomerular hypertension and chronic ischemia, two complementary mechanisms recognized as major drivers of progressive nephron loss and CKD progression [13, 15, 16].

These findings are in agreement with previous biopsy‐based studies in IgA nephropathy, in which intrarenal vascular lesions, including arteriolosclerosis and arterial intimal fibrosis, were independently associated with accelerated renal function decline and progression to kidney failure [17]. Likewise, vascular injury has consistently emerged as an independent determinant of adverse renal outcomes across different forms of CKD, supporting the concept that chronic vascular remodeling provides prognostic information beyond glomerular lesions alone [15, 18].

In line with this concept, biopsy studies have demonstrated that nephroangiosclerotic vascular changes are frequently observed across different glomerular diseases and cannot be considered merely a consequence of hypertension, but rather a shared histopathological feature of chronic kidney injury [19, 20, 21]. Accordingly, our findings suggest that arteriolosclerosis represents an integrated marker of cumulative microvascular damage, identifying patients at increased risk of progressive kidney dysfunction beyond conventional clinical markers. The persistence of renal function decline despite a significant reduction in proteinuria further supports the hypothesis that chronic vascular injury constitutes an additional pathway of CKD progression, operating in parallel with glomerular inflammation and only partially influenced by therapies primarily targeting proteinuria.

Another important finding of the present study is the independent prognostic value of arteriolosclerosis. In the multivariable model, arteriolosclerosis emerged as the only variable independently associated with the composite renal and cardiovascular outcome after adjustment for age, baseline eGFR, and proteinuria. These findings suggest that chronic vascular lesions provide incremental prognostic information beyond conventional clinical markers and may identify patients at increased risk of adverse cardiorenal events.

Our findings are consistent with those of the Boston Kidney Biopsy Cohort, in which arterial and arteriolar sclerosis independently predicted kidney disease progression even after adjustment for baseline eGFR, proteinuria, and clinicopathological diagnosis, highlighting the prognostic value of vascular pathology beyond traditional clinical variables [22]. In light of the 2021 KDIGO Blood Pressure Guideline [6], which recommends a standardized office SBP target of <120 mmHg in most adults with CKD when tolerated, our findings provide histopathological support for intensive blood pressure control. The association between higher SBP, arteriolosclerosis, and adverse cardiorenal outcomes suggests that chronic vascular lesions may identify patients in whom achieving lower SBP targets is particularly relevant to limit progressive vascular and renal injury.

Similarly, Ruan et al. demonstrated that arteriolosclerosis and arterial intimal fibrosis were independently associated with adverse renal outcomes in patients with IgA nephropathy [17]. Extending these observations, our study suggests that the prognostic significance of arteriolosclerosis also applies to a broader population of patients with biopsy‐proven GN and encompasses both renal and cardiovascular outcomes.

From a clinical perspective, these findings support the systematic evaluation of vascular lesions during routine histopathological assessment. The identification of arteriolosclerosis may improve risk stratification by providing prognostic information complementary to traditional clinical markers, thereby helping to identify patients who may benefit from closer follow‐up and more intensive management of blood pressure and cardiovascular risk factors.

Our findings should also be interpreted in the context of the evolving recommendations for blood pressure management in CKD. While recent international guidelines differ slightly in treatment thresholds and SBP targets, they consistently emphasize intensive blood pressure control and a personalized approach aimed at maximizing cardiorenal protection. In particular, the 2021 KDIGO guideline recommends a standardized office SBP target <120 mmHg when tolerated, whereas the 2024 ESC and the 2025 AHA/ACC guidelines advocate similarly intensive SBP control in most patients with CKD. Our findings provide histopathological support for these recommendations by demonstrating that higher office SBP is associated with chronic vascular remodeling and that arteriolosclerosis independently identifies patients at increased risk of adverse cardiorenal outcomes [23].

This study has some strengths. First, it combined standardized OBPM with detailed histopathological assessment in a well‐characterized cohort of patients with biopsy‐proven GN, allowing us to investigate the relationship between blood pressure, chronic vascular lesions, and long‐term renal function. Second, the longitudinal follow‐up enabled the evaluation of changes in blood pressure, proteinuria, and renal function over time, as well as the prognostic significance of vascular lesions for renal and cardiovascular outcomes.

Several limitations should also be acknowledged. The retrospective single‐center design limits the ability to establish causal relationships and may reduce the generalizability of our findings. The relatively small sample size and the limited number of outcome events may have reduced statistical power and contributed to the wide confidence intervals observed in the multivariable analysis. In addition, blood pressure assessment was based on OBPM rather than ambulatory blood pressure monitoring, which may have underestimated the contribution of masked or nocturnal hypertension. Therefore, larger prospective multicenter studies are warranted to validate these findings and determine whether integrating histopathological vascular lesions with blood pressure assessment improves risk stratification and guides blood pressure targets in patients with GN.

## 5 Conclusion

In conclusion, our findings identify arteriolosclerosis as a key histopathological marker linking elevated SBP to adverse renal and cardiovascular outcomes in patients with biopsy‐proven GN. The combination of standardized OBPM and histopathological assessment of vascular lesions may refine cardiorenal risk stratification beyond conventional clinical markers and provide a rationale for individualized blood pressure management.

## Author Contributions

Antonietta Gigante contribute to the conception, writing and supervision. Alice Trombacco, Giulia Galanti, Georgia Sorato and Rosa Cascone contribute for validation, investigation, and data curation. Chiara Pellicano and Edoardo Rosato substantial contributions analysis and interpretation of data for the work; Konstantinos Giannakakis contribute to methodology. Rosario Cianci contribute to conceptualization.

## Funding

The authors have nothing to report.

## Conflicts of Interest

The authors declare no conflicts of interest.

## Clinical Study Registration Statement

This study is an observational cohort study and does not involve interventional clinical trials; therefore, clinical study registration was not required.

## Data Availability

The data that support the findings of this study are available from the corresponding author upon reasonable request.
